# Spiritual awakening. Substance abuse, dual pathology

**DOI:** 10.1192/j.eurpsy.2024.1014

**Published:** 2024-08-27

**Authors:** M. J. Gordillo Montaño, L. Rodriguez Rodriguez, C. Pérez Aparicio, S. V. Boned Torres

**Affiliations:** ^1^Hospital Can Misses, Eivissa, Spain

## Abstract

**Introduction:**

Kambó is considered an “ancestral medicine” by the indigenous tribes of the western region of the Amazon.

**Objectives:**

Through this clinical case, the aim is to present the particularities of the symptoms and management of patients with consumption of not so common substances, such as Kambo or salvia divinorum, as well as the evolution that will occur in a patient with a previous diagnosis of a Depressive Episode.

**Methods:**

We present the case of a 23-year-old male, Gestalt therapy student. History of tobacco, THC, and recent use of salvia divinorum and Kambo. He began follow-up by psychiatry in a private setting three years ago due to a severe depressive episode, having required treatment with antidepressants, antipsychotics and benzodiazepines, and having been triggered by a serious assault. The episode is resolved and follow-up is discontinued. Family history of depressive syndrome and suicide.

He resumed contact through the Emergency Department, requiring hospital admission due to symptoms compatible with a manic episode with psychotic symptoms. It begins with behavioral alterations and global insomnia that are related to the consumption of some substance, initially unknown to them, making the skin lesions they presented suspect the consumption of kambo.

**Results:**

We assess the risk of consuming these substances, which are sometimes used as alternative therapies, and especially in this type of patient, who is more vulnerable and perhaps seeks a way out of the problems they present.

**Conclusions:**

In our case, it triggered a manic episode with psychotic symptoms, which consisted of delusional ideation of mystical content accompanied by auditory hallucinations. The episode took about a month to subside, despite treatment. Subsequently, there have been more episodes with similar characteristics, and they have not been associated with the consumption of kambó, but have been linked to the consumption of “natural medicinal substances.”

**Disclosure of Interest:**

None Declared

